# Acute Increase of Children's Conjunctivitis Clinic Visits by Asian Dust Storms Exposure - A Spatiotemporal Study in Taipei, Taiwan

**DOI:** 10.1371/journal.pone.0109175

**Published:** 2014-10-27

**Authors:** Lung-Chang Chien, Yi-Jen Lien, Chiang-Hsin Yang, Hwa-Lung Yu

**Affiliations:** 1 Division of Biostatistics, University of Texas School of Public Health at San Antonio Regional Campus, San Antonio, Texas, United States of America; 2 Department of Bioenvironmental Systems Engineering, National Taiwan University, Taipei, Taiwan; 3 Department of Health Care Management, National Taipei University of Nursing and Health Sciences, Taipei, Taiwan; Medical College of Soochow University, China

## Abstract

Adverse health impacts of Asian dust storms (ADS) have been widely investigated and discussed in respiratory disease, but no study has examined the association between ADS events and their impact on eye diseases, especially in children. The impact of ADS events on the incidence of children's conjunctivitis is examined by analyzing the data from children's clinic visits registered in the 41 districts of Taipei area in Taiwan during the period 2002–2007. The structural additive regression modeling approach was used to assess the association between ADS events and clinic visits for conjunctivitis in children with consideration of day-of-the-week effects, temperature, and air quality levels. This study identifies an acute increase in the relative rate for children's conjunctivitis clinic visits during ADS periods with 1.48% (95% CI = 0.79, 2.17) for preschool children (aged <6 years old) and 9.48% (95% CI = 9.03, 9.93) for schoolchildren (aged ≥6 years old), respectively. The relative rates during post-ADS periods were still statistically significant, but much lower than those during ADS periods. The spatial analysis presents geographic heterogeneity of children's conjunctivitis clinic visits where higher relative rates were more likely observed in the most populated districts Compared to previous ADS studies related to respiratory diseases, our results reveals significantly acute impacts on children's conjunctivitis during ADS periods, and much influence on schoolchildren. Vulnerable areas were also identified in high density population.

## Introduction

Asian dust storm (ADS) events, severe wide-spread disasters that spawn from Mongolia and North China, cause many diseases including respiratory diseases, cardiovascular diseases, asthma, chronic obstructive pulmonary disease, pneumonia, and stroke. The health outcomes of these diseases range from death to various stages of illness that may require hospital admissions, emergency room visits, and clinic visits [1]–[7]. ADS events diversely impact human health primarily due to a rapid elevation of concentrated levels of air pollutants over a large scale area in a short period of time [8]. Inhalation of air pollutants during these storms has been implicated as the leading cause of adverse health effects. However, eye exposure to direct contact with air pollutants during these storms and the subsequent development of conjunctivitis has rarely been discussed.

The eyes are particularly vulnerable and highly sensitive to direct contact with air pollutants due to the dense neuronal innervations on the ocular surface [9]. For eye-related diseases which are highly correlated with exposure to air pollutants, conjunctivitis is the most significant Conjunctivitis, defined as an irritation or an inflammation of the conjunctiva, usually elicits symptoms of eye irritation and itching. Previous research has established an association between the development of conjunctivitis and the ambient levels of outdoor air pollution or airborne allergens, such as fungal spores and pollen grains [10]–[12]. Indoor air pollution, such as the cooking smoke from the combustion of biomass (e.g., charcoal, wood, and dung), has also been investigated as a causal agent in the development of conjunctivitis and other eye diseases [13]. ADS events are characterized by extremely high concentrations of air pollutants and allergens compared to the concentrations of these same pollutants and allergens during general periods. Thus, the development of conjunctivitis as a result of exposure during ADS events would be highly likely [14]. However, epidemiological research on the ADS effects to eye health is still rare,, and has not yielded any significant findings [15].

Children, in particular, are very susceptible to the adverse effects of airborne exposures because their immune systems are not as fully developed as those of adults [16]. Previous studies focused on the adverse effects of air pollution on children's health in terms of both short-term [17]–[21] and long-term exposure [22]–[24], These pediatrics researches have been centered on investigating pulmonary and lung dysfunction, asthma and respiratory symptoms, and pneumonias, especially for the case of the studies regarding to the influence of ADS events In addition, findings regarding the health impact of ADS events are commonly uncertain because of limited health observations [25]. In the other words, previous ADS and children's health studies have seldom focused on conjunctivitis and analyzed large scale populations of children observed over long periods of time and at varied locations [26]. Due to the availability of nationwide health insurance data in Taiwan, it allows the population-based analysis to reveal the epidemiological linkages between ADS events and the diseases of concern, e.g. conjunctivitis [27], [28].

The main purpose of this study was to investigate the spatiotemporal influence of ADS events on the development of conjunctivitis in children by using a large, population-based database taken from daily clinic visits registered in hospitals and clinics in Northern Taiwan from 2002–2007. Specifically, three main questions were addressed: (1) whether there is an increase in children's clinic visits for conjunctivitis during ADS events and for one week after the storm; (2) whether preschool children are more vulnerable than school children to the impact of ADS events as they relate to the development of conjunctivitis; and (3) whether geographic location plays a role in the impact of ADS events on children's conjunctivitis clinic visits.

## Materials and Methods

Ethic statement: The data were obtained from National Health Research Institute of Taiwan (NHRI). All of the data request should be approved by IRB of NHRI. The approval number of this study is NHIRD-102-097. Due to concerns regarding personal confidentiality, all individually identifiable health information (e.g., personal identification or hospital identification number) is encrypted prior to release.

### Children's clinic visit data

This study retrieve the children's clinic visit data from Taiwan's National Health Insurance (NHI) database which covers the claim data of over 99.6% of the inhabitants of the entire Taiwan, including both emergency and ambulatory visits for various diseases and diagnosis. The database uses International Classification of Diseases, Ninth Revision, Clinical Modification (ICD-9-CM) classification codes to record cause-specific data. Due to concerns regarding personal confidentiality, all individually identifiable health information (e.g., personal identification or hospital identification number) is encrypted prior to release from Bureau of NHI. This study retrieved patients aged ≤14 years with recorded conjunctival disorders (ICD-9: 372). Because children older than 6 years old in Taiwan receive compulsory education, this population was categorized into a preschool group for <6 years-old and a schoolchildren group aged ≥6 years old. This population-based data also contained space-time information for any clinic and hospital visits of children with the diagnosis of conjunctivitis in Taipei City and New Taipei City from 2002–2007. These visits included both ambulatory and emergency room encounters.

### Study Area

**Figure 1:**
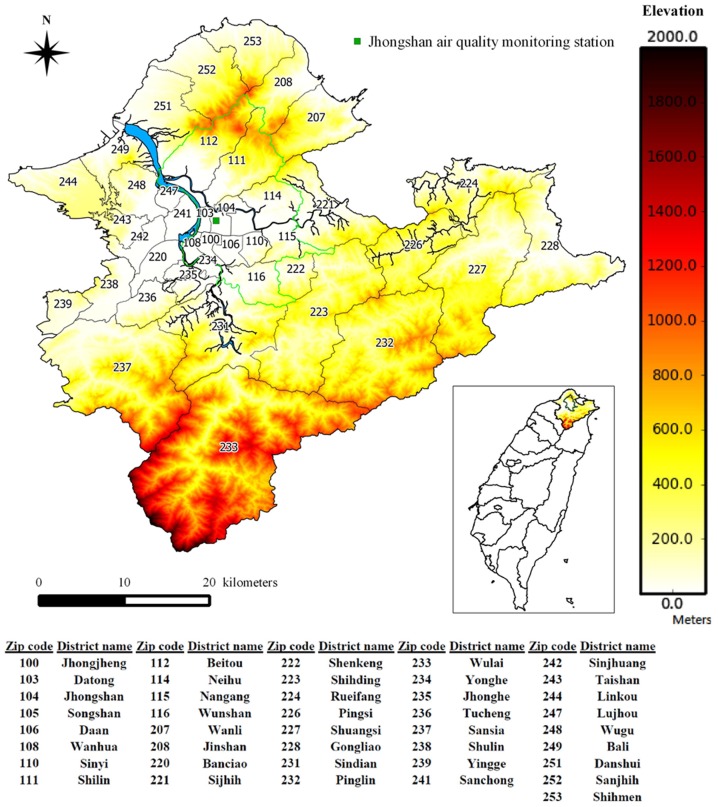
Map of Taipei City (surrounded by green boundaries) and New Taipei City, along with the map of their geographical locations in the Taiwan Island. The district names are denoted by their zip codes with the names shown at the table below. The Jhongshan air quality monitoring station is highlighted by a green square.

Taipei has more than six million citizens within an area of approximately 2324.37 km^2^. Taipei is best described as a geographic location in Northern Taiwan that contains two administrative regions, Taipei City and New Taipei City. Among them, New Taipei City surrounds Taipei City. The northern boundary is the coastline for to the Taiwan Straits and the Pacific Ocean. This northern coastline border offers no geographic barrier to ADS events that sweep in from the north. In addition, Taipei City has a characteristic basin type of topography that is geographically bounded by three different Mountains to the north, the west, and the southeast. The basin topography reduces air diffusion and increases air pollutant concentrations. According to insurance administrative divisions, a total of 41 districts are contained in the study area with 12 districts for Taipei City and 29 districts for New Taipei City, as shown in Figure 1.

### Asian dust storm and air pollution data

A total of 30 ADS events occurring from 2002–2007 were identified by the Taiwan Environmental Protection Agency (TWEPA), during a total of 90 days (Table 1). The TWEPA defines the criteria to determine the incidence of a ADS event, including performing a dust storm track nowcasting along with Taiwan Central Weather Bureau, interpreting remote sensing data, and observing the PM_10_ level at the four ADS indicator stations, i.e., Matsu, Wanli, Guanyin, and Yilan. If forecasting results reveal a high probability that ADS events are headed toward Taiwan, the TWEPA will issue warnings when at least one of the four indicator stations measure PM_10_ concentrations >100 µg/m^3^ [29]. For the analyses purposes the study period from 2002 to 2007 was divided into three sub-periods. First, the time period of the actual ADS event (Table 1) is defined as the ADS period. Second, the time period up to and including 7 days after an ADS event has ended is defined as the post-ADS period. Third, all the other days in the study period are defined as the other period. In addition to the ADS data, PM_10_ concentrations have been regularly monitored at TWEPA stations across Taiwan since 1994. Both the PM_10_ concentrations and temperature measurements used in our analysis were based on the daily observations at the Jhongshan monitoring station located in the most populated area of Taipei City (see Figure 1).

**Table 1:** Dust storm events in Taiwan, 2002–2007.

Year	Date	# of events	# of days
2002	2/11-2/12, 3/6-3/9, 3/23-3/24, 3/31-4/1, 4/8-4/15, 4/17-4/19	6	21
2003	2/18-2/19, 2/23-2/25, 3/6-3/9, 3/25-3/30, 4/25-4/28	5	19
2004	1/1-1/4, 1/13-1/14, 1/21-1/22, 1/24-1/25, 2/6-2/12, 2/14-2/16, 2/26-2/27, 3/3-3/7, 4/2-4/4	9	30
2005	3/18-3/19, 11/29-11/30, 12/21-12/22	3	6
2006	3/19-3/20, 3/29-3/30, 4/20-4/21	3	6
2007	1/28-1/29, 4/2-4/3, 4/17-4/18, 12/30-12/31	4	8

### Statistical Modeling Analysis

We analyzed the spatiotemporal pattern of children's conjunctivitis clinic visits in the 41 study districts from 2002 to 2007 using a structured additive regression (STAR) model. The count data for clinic visits followed a Poisson distribution, and can be modeled via a Poisson model framework taking into account both linear and nonlinear explanatory variables, i.e.,
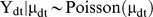
Y_ct_ is the number of children's conjunctivitis clinic visits in district d (d = 1, 2,…, 41) at day t (t = 1, 2,…, 2191).
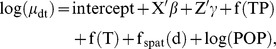
where μ_dt_ is the expected value of Y_dt_. The β is a vector of categorical fixed effects X = [I_MON_, I_TUE_, I_WED_, I_THUR_, I_FRI_, I_SAT_, I_ADS_, I_POST-ADS_]′ corresponding to six dummy variables for day-of-the-week (DOW) from Monday to Saturday plus two dummy variables for the ADS period and post-ADS period. Theoretically, DOW variables are mainly used to adjust for short-term temporal autoregressive correlations. From a practical standpoint, we are able to investigate the peak number of children's conjunctivitis clinic visits within one week of an ADS event which reflects a special attribute of medical service in Taiwan. Two ADS variables can be used to evaluate the impact of ADS on children's conjunctivitis clinic visit during the ADS period and the post-ADS period compared to other period. The γ is the vector of continuous confounders Z, which contains at least one of five air pollutants (CO, NO_x_, O_3_, PM_10_, and SO_2_). Two nonlinear smoothing functions for calendar time T and for daily mean temperature TP applied a B-spline with a second order random walk penalty [30], [31] to adjust for long-term temporal trends and the effects of weather. The spatial function f_spat_(d) is defined by a structured spatial effect using the Markov random fields [32] with a normally distributed conditional autoregressive prior:
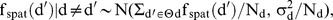
where the denominator N_d_ in both the mean and the variance is the number of neighboring districts adjacent to district d, and θ_d_ is a subset of neighbors of district d. The variance σ^2^_d_ and unknown smoothing parameters are estimated simultaneously with hyper-priors following an inverse Gamma distribution IG(0.001, 0.001). Last, the term log(POP) is the offset defined by the logarithm of the district-level population of children approximated by multiplying the proportion of children in Taipei by the total population of each district.

The STAR model's estimates are calculated according to an empirical Bayes influence that utilizes a restricted maximum likelihood method. This is a surrogate of the maximum likelihood method designed to avoid the loss of degrees of freedom and to prevent biasing estimated coefficients toward zero [33], [34]. Model selection for choosing the best combinations of air pollutants utilized the Akaike Information Criterion (AIC) [35]. Specifically, seven one-pollutant models were fitted for selecting significant positive estimated coefficients of air pollutants. Then, several two-pollutant and three-pollutant models were fitted retaining the significant air pollutants from one-pollutant models. Only the models with positively significant air pollutants were included in the selection to determine the final model according to the smallest AIC separately for preschool children and schoolchildren. In these models, the estimated coefficient of this fixed effect can be explained by the percentage change in the relative rate (RR) of children's conjunctivitis clinic visits compared to the reference level. The spatial function can be interpreted by the RR in district ‘d’ compared with the mean value for the population as a whole and accounting for spatial autocorrelations by taking a logarithm of it [36]. The spatial effect in each district has a posterior distribution which can be used to generate a 95% credible interval (CI) in order to determine the significance of each spatial estimate according to the 95% posterior probability. For a more meaningful presentation, the spatial effect can be categorized into: (1) a strictly positive effect if 95% of the posterior distribution is above one; (2) a strictly negative effect if 95% of the posterior distribution is below one; (3) a non-significant effect if 95% of the posterior distribution contains one. Maps for the estimated spatial effects with corresponding 95% posterior probability were constructed to highlight the spatial pattern of children's conjunctivitis clinic visits. Data were cleaned and managed by using SAS v9.3 software (SAS Institute Inc., Cary, NC), and the spatiotemporal data analyses were accomplished by the BayesX v2.01 software package [37].

## Results

**Figure 2:**
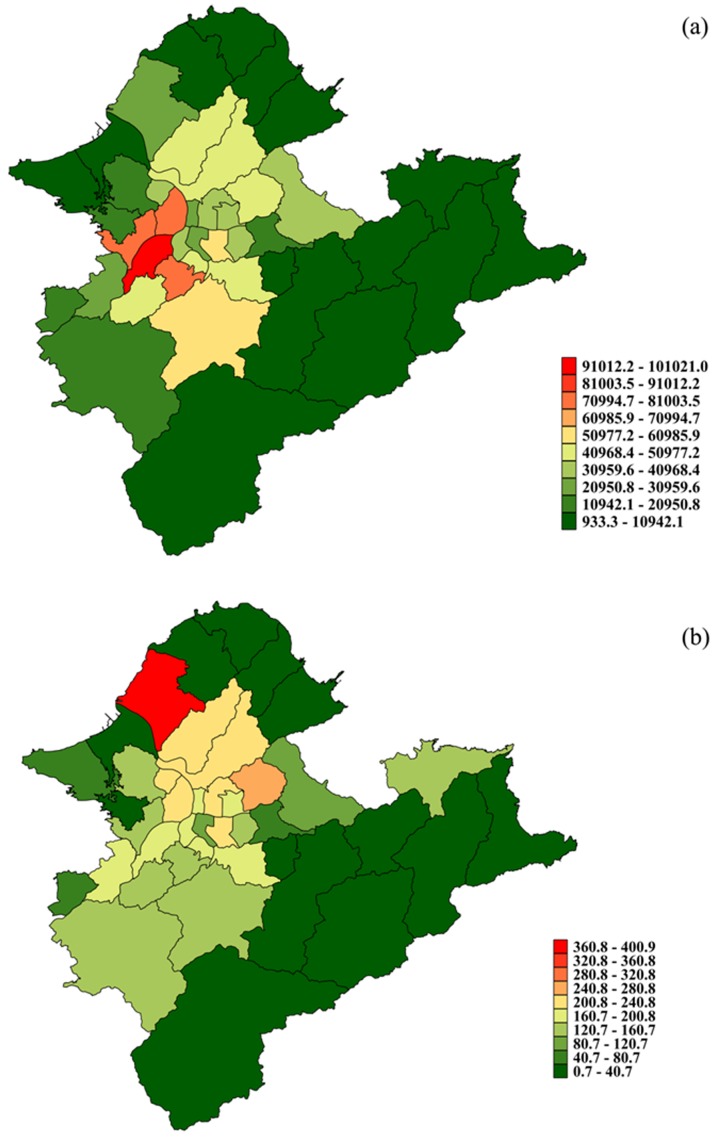
Maps of (a) spatial distribution of the average children population and (b) crude daily children's conjunctivitis clinic visit rate per 100,000 children population in Taipei, 2002–2007.

Figure 2 depicts the crude rate of daily children's conjunctivitis clinic visits among the 41 districts in Taipei, clearly demonstrating that the crude clinic visit rate was not uniformly distributed across the study area. By city, the crude rate per 100,000 children in Taipei City ranged from 47.98 to 266.28, but there was a wider range from 0.72 to 400.89 in New Taipei City, which means that the geographic disparity of children's conjunctivitis morbidity there may be larger than that in Taipei City. Table 2 documents that the daily average measurements of O_3_ and PM_10_ were elevated during the ADS period. Their averages decreased during the post-ADS period, but were still higher than levels during other period. The other air pollutants had decreased average measurements from the other period through the ADS period, but these were increased during the post-ADS period.

**Table 2:** Average measurement of air pollutants during ADS periods, post-ADS periods and Other periods in Taipei, Taiwan, 2002–2007.

Air pollutant	ADS period		Post-ADS period		Other period		P-value††
	Mean	Std†	Mean	Std†	Mean	Std	
CO (ppm)	0.82	0.30	1.01	0.33	0.88	0.32	<.0001
NO_x_ (ppb)	45.50	17.43	60.10	23.38	49.77	19.51	<.0001
O_3_ (ppb)	25.13	7.40	20.60	8.00	18.99	8.29	<.0001
PM_10_ (µg/m^3^)	81.11	35.41	63.04	25.44	53.32	22.51	<.0001
SO_2_ (ppb)	3.91	2.03	4.17	2.28	4.35	2.08	<.0001

† Std = standard deviation.

†† P-value was determined by the ANOVA test with null hypothesis H_0_: μ_ADS_ = μ_post-ADS_ = μ_other_.

**Figure 3:**
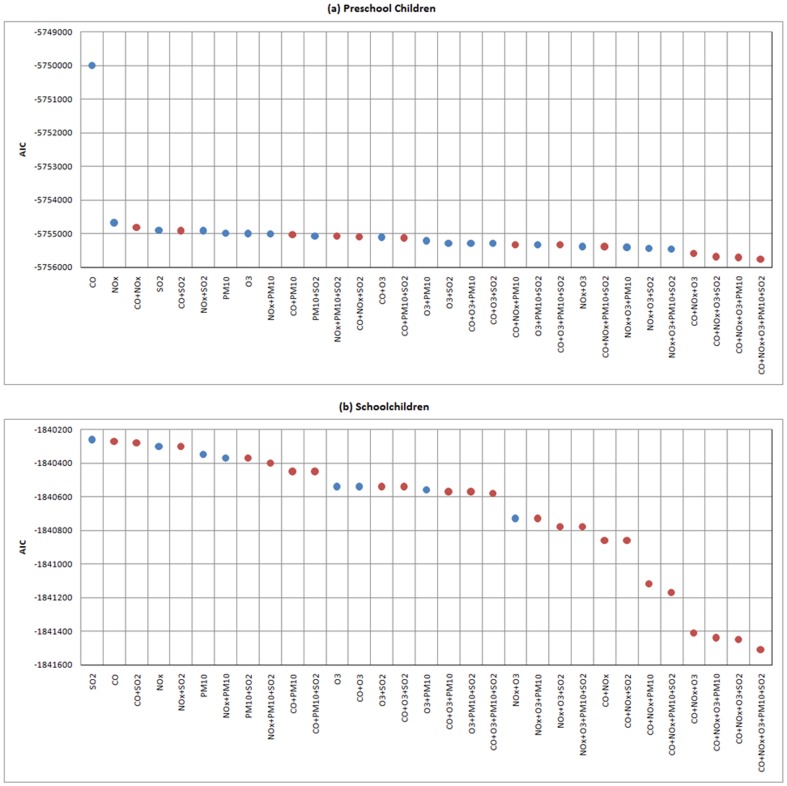
The model selection for (a) preschool children and (b) schoolchildren, where blue dots represent models with all significantly positive estimates for air pollutants, and red dots represent models with at least one non-significant or significantly negative estimate for air pollutants. Our final models were only considered among blue dots.

Figure 3 gives ranked values of AIC for the fit and complexity of the selected models. It suggests that among those 31 models, the AIC was when more air pollutants were included, and the smallest AIC appeared in the five-pollutant models. Models shown in red dots in Figure 3 were not considered because they had non-significant or significantly negative estimates even if they had a smaller AIC. Therefore, the four-pollutant model NO_x_+O_3_+PM_10_+SO_2_ for preschool children and the two-pollutant model NOx+O3 for schoolchildren were finally determined as the best final models.

Table 3 displays the percentage change of RR for children's conjunctivitis clinic visits, suggesting: (1) Compared to Sunday, a relatively higher percentage of RR increase for preschool children's and schoolchildren's conjunctivitis clinic visits was more likely happened on a Monday, a Wednesday, and a Saturday; (2) schoolchildren had a higher increased percentage change in RR than preschool children for conjunctivitis clinic visits during ADS periods; (3) a lasting positive impact (i.e., an increased percentage change of RR) during post-ADS periods was noted in schoolchildren; and (4) both NO_2_ and O_3_ were significantly and positively associated with children's conjunctivitis clinic visits. Specifically, the strongest DOW effect appeared on Monday for preschool children with a 47.90% (95% CI = 47.33, 48.47) elevated RR and on Saturday for schoolchildren with a 29.20% (95% CI = 28.67, 29.72) elevated RR, compared to Sunday, a day on which the clinics are closed. During the ADS period, the RR significantly increased 1.48% (95% CI = 0.79, 2.17) for preschool children's conjunctivitis clinic visits, while the percentage increased over 6-fold to 9.48% (95% CI = 9.03, 9.93) for schoolchildren. This impact did not extend through the post-ADS period for preschool children and the effect showed a negative decline with a −2.16% (95% CI = −2.68, −1.63) RR. However, schoolchildren were still significantly affected during the post-ADS period with a 2.32% (95% CI = 1.98, 2.66) elevation in the RR for conjunctivitis clinic visits. Moreover, air pollutants, NO_x_ and O_3_, also had a stronger influence on conjunctivitis clinic visits for schoolchildren than for preschool children. Interestingly, O_3_ had a stronger impact than NO_x_ for both groups.

**Table 3:** Percentage change of relative rate in children's conjunctivitis clinic visits.

	Preschool children		Schoolchildren	
Variable	%	95% CI	%	95% CI
Day-of-week†				
Monday	47.90	(47.33, 48.47)	26.75	(26.41, 27.10)
Tuesday	6.91	(6.44, 7.38)	−7.13	(−7.41, −6.84)
Wednesday	14.43	(13.95, 14.92)	26.82	(26.48, 27.17)
Thursday	3.18	(2.72, 3.63)	−13.23	(−13.50, −12.96)
Friday	6.42	(5.96, 6.89)	4.17	(3.87, 4.47)
Saturday	29.20	(28.67, 29.72)	74.70	(74.29, 75.12)
ADS episode†				
ADS period	1.48	(0.79, 2.17)	9.48	(9.03, 9.93)
Post-ADS period	−2.16	(−2.68, −1.63)	2.32	(1.98, 2.66)
Air pollutant (interquartile range)††				
NO_x_ (22.16 ppb)	1.63	(1.34, 1.92)	3.49	(3.34, 3.66)
O_3_ (11.22 ppb)	3.26	(2.93, 3.59)	6.21	(6.02, 6.40)
PM_10_ (29.88 µg/m^3^)	0.42	(0.10, 0.74)	–	–
SO_2_ (2.63 ppb)	1.19	(0.87, 1.51)	–	–

† The reference level of day-of-week and ADS episode are Sunday and the other days, respectively.

†† The estimate was presented by the increase percentage of relative rate for an interquartile range change in air pollutants, calculated from [exp(interquartile range×estimated coefficient)−1]×100%.

**Figure 4:**
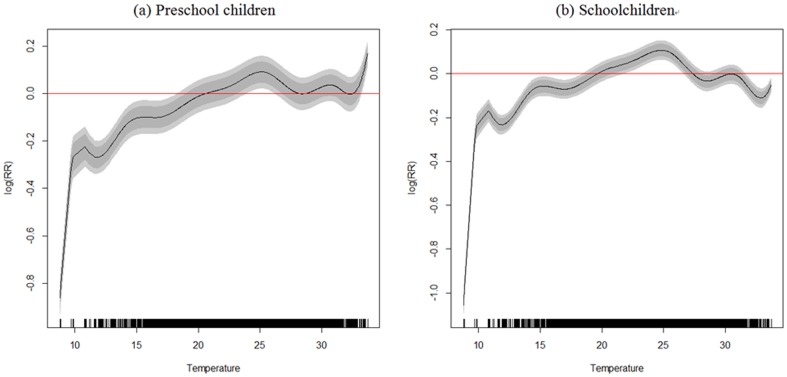
Temperature smoother for (a) preschool children's and (b) schoolchildren's conjunctivitis clinic visits. Dark grey and light grey colors represent 80% and 95% credible intervals, respectively.

The influences of time and temperature on children's conjunctivitis clinic visits demonstrated a greater RR during summers when the temperature was hotter (see Figure 4). In particular, in preschool children there was a consistent significantly positive association between conjunctivitis clinic visits and temperature, especially when the temperature increased from 23.92°C to 26.19°C. The greatest RR = 1.18 (95% CI = 1.12, 1.25) appeared at an extremely hot temperature (33.76°C). For schoolchildren, a significant RR >1 for conjunctivitis clinic visits happened between temperatures of 21.80°C to 26.61°C, and the greatest RR of 1.11 (95% CI = 1.07, 1.16) occurred at a temperature of 24.84°C.

**Figure 5:**
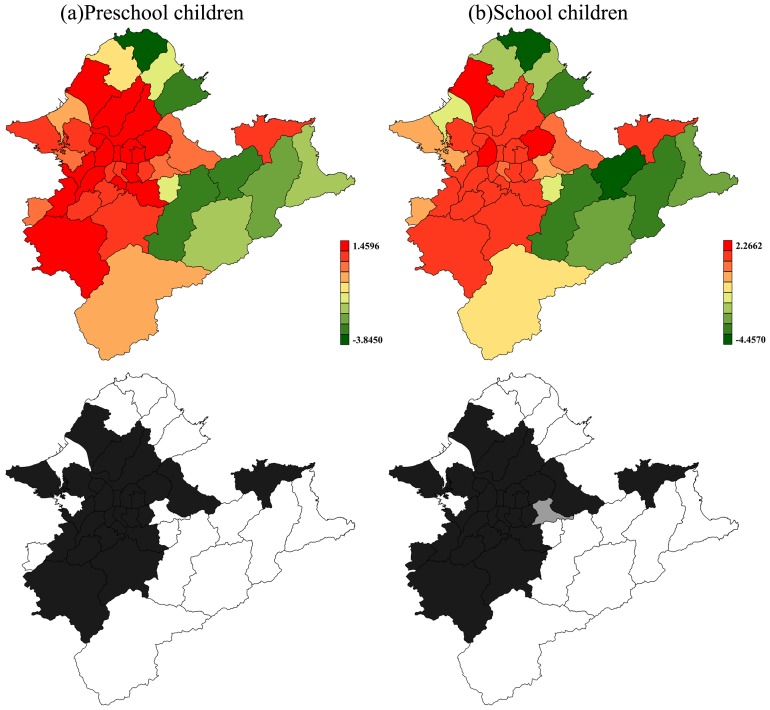
Maps of spatial effects (upper) with corresponding significance (lower) for (a) preschool children's and (b) schoolchildren's conjunctivitis clinic visits in Taipei, Taiwan, 2002–2007, presented by RR. In upper plots, the red color signifies higher RR, and the green color signifies lower RR. In lower plots, black and white areas demonstrate districts where the RR is statistically greater than 1 and smaller than 1, respectively. Grey areas demonstrate no significant difference.

Figure 5 depicts the distribution of spatial effects attributed to children's conjunctivitis clinic visits across 41 districts. It suggests that the significant RR >1, appeared in the more populated areas which included 11 of the 12 districts in Taipei City and 16 of the 29 districts in New Taipei City. Districts near the mountains in the southwest and the coastlines in the north exhibited a significant RR<1. The two maps of 95% posterior probability in the two children's groups were almost identical, except for the Nangang District. Moreover, out of all the 27 districts with a significantly elevated RR, the average RR was 2.70 for preschool children and 3.80 for schoolchildren. The Danshui District displayed the strongest spatial effect contributing to conjunctivitis clinic visits for both children's groups. Comparisons between the two children's groups reveal that 25 of the 41 districts had larger RRs for schoolchildren's conjunctivitis clinic visits than for preschool children's conjunctivitis clinic visits. Of these districts, 11 were in Taipei City and 14 were in New Taipei City.

## Discussion

As extreme weather events, ADS bring a higher concentration of air pollutants to Taiwan, and lead to increased risk of disease incidence and hospitalizations. This risk has been documented to be heterogeneous across space and time [38]. Previous studies emphasized the ADS impact on upper respiratory tract diseases, pneumonia and cardiovascular diseases [4], [5], [39]. Thus far, no significant finding related to the impact of ADS on the development of conjunctivitis had been documented [15]. This may have been due to inadequate sample sizes and the lack of inclusion of spatiotemporal associations leading to insufficient power and possible biases [15]. This research reveals the statistically significant associations between ADS exposure and children's eye health. Based on the large Taiwan National Health Insurance dataset, this study has a lower risk of containing biased estimates resulting from sampling limitations. In addition, spatial and temporal autocorrelations relating to health outcomes are also controlled in this analysis. Therefore, this study reliably provides objective findings based on a large sample size that also covers a broad geographic area during the defined study period and utilizes a well-established statistical modeling approach.

In this study, we found a significant association between ADS exposure and conjunctivitis in both preschool children and schoolchildren. Previous findings showed a similar association between ADS exposure and respiratory diseases in children [26]–[28]. Importantly, this study notes that increased impacts of children's conjunctivitis occurred during ADS periods; while increased impacts of children's respiratory illnesses occurred during post-ADS periods [26]–[28]. Moreover, during the ADS period, schoolchildren had a 6.41 times higher percentage of RR for conjunctivitis clinic visits than preschool children. Previous research has shown that the increase of PM concentration outdoors during ADS events is significantly higher than the increase of PM concentration indoors [40]. While the prevalent seasons for ADS events, winter and spring, overlap the two academic semesters, there is not an ADS-related class suspension policy in Taiwan. As a result, schoolchildren have a higher chance of outdoor exposures during ADS events, going to and from school, than preschool children. Schoolchildren may also have more outdoor activities than preschool children. This exposure most likely explains a greater increase in the conjunctivitis clinic visit rates for schoolchildren. The influence of schoolchildren and preschool children had opposite directions during post-ADS period where schoolchildren still positively affected by ADS with an increased RR% (2.32%) while preschool children's RR% became negative (−2.16%). This situation may be explained by the distinct behavior patterns between the two groups of children. Schoolchildren, as compared to preschool children, have a greater opportunity for ADS exposures during and after the ADS events because they experience more outdoor activities. The negative RR% in preschool children could be interpreted by a protective mentality of parents who feel uncertainty about when the ADS finishes, and may bring children outside to look for eye doctors instead of directly using medicines which can be bought from pharmacy stores without prescriptions.

In order to delineate the health impact of ADS events in regards to conjunctivitis, the short-term and long-term temporal patterns of conjunctivitis clinic visit rates were also analyzed. Among them, the day-of-week pattern is an important confounding factor for clinic visits, and this is true for other diseases as shown in previous studies [26], [27]. In Taiwan, major medical services in both hospitals and clinics are closed from Saturday afternoon until Monday morning. Therefore, there is a strong incentive to visit clinics on Saturday (before the weekend effect) and Monday (after the weekend effect). The day-of-the-week clinic visit pattern observed in this study is quite consistent with the medical care-seeking pattern that has resulted under the current national health care delivery system in Taiwan. Moreover, the highly elevated relative rates on Monday essentially accounts for those patients unable to seek medical treatments from Saturday through Sunday. In the case of children, who are incapable of accessing clinic care independently and are dependent on a parent's working schedule which allows for only nighttime availability, this may be especially true. In addition, while the implementation of NHI in Taiwan increases the accessibility of medical services with relatively low co-payments, prescription medications can only be issued for a three day period [41]. After three days, the patient must see the physician again to obtain more medication if necessary. This prescription regulation may induce higher clinic visit rates on Wednesdays as noted in this study and in previous studies [27].

This study reveals a geographic heterogeneity for conjunctivitis incidence as verified by studying the documented clinic visits. Notably, there is a decreasing trend in relative rates from the northwestern coastal districts to the urban areas. In particular, the Danshui District has a greater RR than its surrounding coastal districts, i.e., the Sanjhih District, Shihmen District, Jinshan District, Wanli District, and Bali District. This result can be attributed to the imbalance of medical resources in the area. Though the northwestern coastal districts are noted for their popular oceanic recreational areas for swimmers and tourists, the numbers of well-equipped hospitals and clinics are much less numerous than in the Danshui District. In addition, the lack of eye departments in coastal area might propel residents to consult the medical center in Danshui district. Convenient transportation is another advantage in that eye clinics in the Danshui District are more accessible than nearby areas. As a result, medical services located in the Danshui District are most often the first choice for the tourists and local residents. Another important factor contributing to the increase in conjunctivitis clinic visits in this district relates to its geographical location. The Danshui District is located near an outlet where the Danshui River flows into the Taiwan Strait and therefore, sits at the opening of the Taipei Basin. Because of this location, wind speeds are generally higher in the Danshui District [42]. Studies have shown that dust and wind are the major irritating factors that often trigger eye diseases, such as conjunctivitis [12], [43], [44]. The distinct features of the spatial function in the STAR model not only control for spatial autocorrelation, but also facilitate the analysis of the geographical heterogeneity in conjunctivitis clinic visit. In addition, short-term and long-term temporal pattern modeling could be performed as in other diseases and health outcomes [26]–[28]. After consideration of the general spatial and temporal patterns of conjunctivitis clinic visit rates and their associations with covariates, ambient pollutants may better differentiate the health impact from ADS exposure on the subsequent development of conjunctivitis.

This study has three main limitations: First, the study period of this research is restricted to only 6 years because of inconsistent disease coding without the use of ICD-9 before 2002 plus a funding shortage after 2007. Second, some potential allergies to conjunctivitis, such as pollen grain and fungal spores, do not have district-level measurements, so this study is unable to include them as confounding predictors. Third, the STAR model has a complicated estimating algorithm in that, sometimes, unknown parameters cannot converge. This is especially when considering pre-ADS periods for 7 days prior to ADS periods or longer post-ADS periods up to 14 days.

## Conclusions

This study demonstrates significantly elevated risks in children's clinic visits due to conjunctivitis in both preschool children and schoolchildren during ADS periods in Taipei, Taiwan. This acute impact produced an increased percentage of RR for clinic visits due to conjunctivitis in schoolchildren during the ADS period, and lasted during the 1 week post-ADS period. In addition, new evidence about the spatial disparities for the adverse effects of ADS events on children's eye health in northern Taiwan was also discovered. Children in all ages are suggested to reduce their overall outdoor exposure time during the ADS period and the post-ADS period, especially for schoolchildren who live in high risk districts. The results and findings of this study should be of particular concern to policy makers or media outlets that are responsible for issuing ADS warnings in advance.
